# Custom total knee arthroplasty with personalised alignment showed better 2‐year functional outcome compared to off‐the‐shelf arthroplasty

**DOI:** 10.1002/ksa.12309

**Published:** 2024-06-17

**Authors:** Nicole Vogel, Raphael Kaelin, Markus P. Arnold

**Affiliations:** ^1^ Practice MEIN KNIE, Hirslanden Klinik Birshof Münchenstein Switzerland; ^2^ Practice LEONARDO, Hirslanden Klinik Birshof Münchenstein Switzerland; ^3^ Faculty of Medicine University of Basel Basel Switzerland

**Keywords:** matched‐pair analysis, patient‐reported outcome measure, patient satisfaction, patient‐specific, personalised alignment, total knee arthroplasty

## Abstract

**Purpose:**

Customised individually made (CIM) total knee arthroplasty (TKA) with personalised alignment is relatively new and evidence is limited. The aim of this study was to compare patient‐reported outcome measures between CIM and off‐the‐shelf (OTS) TKA patients in a matched‐pair analysis with a 2‐year follow‐up.

**Methods:**

In this single‐centre, prospective cohort study, propensity score matching was performed on 51 CIM and 51 OTS TKA. Data were measured at baseline, at 4 months, 1 and 2 years and included the Forgotten Joint Score (FJS‐12), the High Activity Arthroplasty Score (HAAS), the Knee injury and Osteoarthritis Outcome Score (KOOS), the EQ‐5D‐3L, the EQ‐Visual Analogue Scale, satisfaction, overall knee improvement, willingness to undergo the surgery again and the Knee Society Score.

**Results:**

At 2 years follow‐up, the FJS‐12 (77 vs. 67, *p* = .058), HAAS (13 vs. 11, *p* < .001), KOOS daily living (92 vs. 86, *p* = .029), KOOS sport (76 vs. 65, *p* = .019), KOOS quality of life (81 vs. 71, *p* = .028) and the EQ‐5D (.95 vs. .90, *p* = .030) were higher for CIM TKA compared to OTS TKA. Satisfaction rate was 92% for CIM TKA and 84% for OTS TKA (*p* = .357). Most patients reported an improvement in the overall knee state (94% CIM and 90% OTS, *p* = .487) and almost all patients would undergo the surgery again (96% CIM and 98% OTS, *p* = .999).

**Conclusion:**

The current study found that CIM TKA patients had better functional outcomes at 2 years. Patient satisfaction was high and not statistically significantly different from OTS TKA patients.

**Level of Evidence:**

Level II, prospective cohort study.

**Abbreviations**
ASA: American Society of Anesthesiologists
BMI: body mass index
CIM: customised individually made
FJS‐12: Forgotten Joint Score
HAAS: High‐Activity Arthroplasty Score
HKA: hip–knee–ankle angle
KL: Kellgren and Lawrence
KOOS: Knee injury and Osteoarthritis Outcome Score
KSS: Knee Society Score
OTS: off‐the‐shelf
PROM: patient‐reported outcome measure
REDCap: Research Electronic Data Capture
SD: standard deviation
TKA: total knee arthroplasty
VAS: Visual Analogue Scale

## INTRODUCTION

Despite improved surgical techniques and treatments, a persistent 20% of total knee arthroplasty (TKA) patients are not satisfied with the outcome [6, 11, 15]. Surgeons are challenged to better understand patient satisfaction and the factors that contribute to it to meet patients' needs. Therefore, great efforts are still being made to further improve outcomes after TKA and to measure these outcomes with patient‐reported outcome measures (PROMs).

The reasons for dissatisfaction are likely multifactorial, but an important surgeon‐related variable is limb alignment [17]. Suboptimal alignment can lead to altered knee kinematics, increased component wear, poor functional outcomes and premature implant failure, suggesting that an optimal alignment technique may improve TKA outcome [3]. Recent improvements in TKA have included a more personalised approach [16], including the use of customised individually made (CIM) TKA and a personalised knee alignment strategy [27]. The morphology of the knee is highly variable, and despite different models and sizes, finding the most appropriate off‐the‐shelf (OTS) implant can be challenging [2, 13]. Implant design and surgical techniques need to better mimic the anatomy and kinematics of the native knee, ultimately providing a forgotten joint [12].

CIM TKAs are designed to anatomically replicate the patient's constitutional morphotype by reproducing limb alignment and restoring joint space by adjusting the offset of the prosthetic condyles [30]. The ORIGIN® prosthesis was launched in 2018 and offers the ability to customise the shape and alignment of the prosthesis [31]. The customisation of the implants allows a positive outcome in terms of alignment and bone surface restoration without the limitations of OTS TKA [31].

The evidence about CIM TKA to date is limited. First results of case series showed promising results with satisfactory clinical outcome and low complication rate [7, 10, 22, 25]. However, they highlight the need for better methodological studies. Comparative studies between CIM and OTS TKA are sparse [35] and not yet available for the ORIGIN® prosthesis. The aim of this study was to compare PROMs between CIM and OTS TKA patients in a matched‐pair analysis with a 2‐year follow‐up. We hypothesised that CIM TKA patients would have better PROMs at 2 years.

## MATERIALS AND METHODS

### Study design, setting and recruitment

This is a single‐centre, observational, prospective cohort study with matched‐pair analyses comparing patients with CIM and OTS TKA. Patients were recruited from two practices in the same hospital. Routinely, all patients scheduled for TKA are asked to complete a set of PROMs [36]. In this study, consecutive patients undergoing primary posterior‐stabilised CIM TKA (ORIGIN® PS, Symbios) or primary cruciate‐retaining OTS TKA (Attune® CR mobile‐bearing, DePuy Synthes) who completed PROMs preoperatively and at 2 years were included. Patients with major re‐operation or revision were excluded.

### Surgical technique

All TKAs were performed between January 2017 and December 2021 by two senior surgeons (M. P. A. and R. K.). All patients underwent the same peri‐ and postoperative anaesthesia and pain management protocol. Both primary TKA systems were used according to the manufacturer's inclusion and exclusion criteria [5, 14]. A medial parapatellar approach without a tourniquet was used. Patients followed the same postoperative rehabilitation protocol, which included immediate full weight bearing on crutches until sufficient muscular stabilisation was achieved.

In CIM TKA, femoral and tibial resections were performed using the custom cutting guides and the femur‐first technique. Soft tissue balance was then assessed using a spacer block. If necessary, the level of resection was adjusted using a custom, single‐use millimetre recut guide. Once all bone surfaces were prepared, trial implants were used to verify correct laxity and stability, after which the definitive implants were cemented. The computed tomography‐based preoperative planning process involves interaction between the engineer and the surgeon. Depending on the constitutional knee phenotype, a validated planning matrix described the individual alignment, and the knees were aligned according to the principles of restricted kinematic alignment to bring them into the so‐called safe zone [5]. By following this procedure, most of the knees did not require any further ligament release beyond the removal of the osteophytes. In larger varus or valgus deformities, the lengthened side (e.g., the lateral side in a varus knee with a hip–knee–ankle angle [HKA] of 168°) may be slightly lax after the bone cuts.

The CIM TKA planning system allows for a maximum of 5° varus or 3° valgus in the resulting HKA. It was avoided to plan towards these possible limits. On the contrary, the aim was to plan the knees towards a neutral alignment, respecting the constitutional phenotype, with a maximum deviation from neutral planning of an HKA of 178° in varus and 181° in valgus knees. The result is a minimal difference between the very limited kinematic alignment of the CIM TKA and the mechanical alignment of the OTS TKA.

OTS TKA was performed with conventional instrumentation aiming at mechanical alignment. The Attune implant is the most commonly used OTS implant in Switzerland [29]. A natural slope and rotation along the grinding marks on the arthritic tibial plateau was aimed for, followed by resection of the tibial plateau. After determining the femoral rotation with the intramedullary balancer, the distal femur was resected first (extension gap), followed by resection of the posterior (flexion gap) and anterior femoral condyles.

Patellar resurfacing was used on an individual and exceptional basis when the primary source of pain was the patellofemoral compartment and the patient declined the risk of a second patellar resurfacing. This was the case in four CIM and three OTS TKA.

### Data analysis and measures

Data were collected at routine visits at baseline, 4 months, 1 and 2 years using Research Electronic Data Capture (REDCap®). Surgeons graded general osteoarthritis by Kellgren and Lawrence (KL) from 0 (no osteoarthritis) to 4 (severe osteoarthritis) [19] and comorbidities by the American Society of Anesthesiologists (ASA) from ASA I (normal healthy) to ASA V (moribund) [1]. Patient characteristics were extracted from medical records.

### Patients completed the following PROMs

− The Forgotten Joint Score (FSJ‐12) ranges from 0 (worst) to 100 (best) points [33].
− The High‐Activity Arthroplasty Score (HAAS) ranges from 0 (worst) to 18 (best) points [32, 34] and is only administered postoperatively.
− The Knee injury and Osteoarthritis Outcome Score (KOOS) with subscales from 0 (worst) to 100 (best) points [26].
− The EQ‐5D‐3L ranges from 0 (worst) to 1 (best), including the EQ‐Visual Analogue Scale (VAS) from 0 (worst) to 100 (best) [9].
− Patient satisfaction on a 5‐point Likert scale, whereby patients were classified as satisfied (very satisfied/satisfied) or not satisfied (neutral/unsatisfied/very unsatisfied).
− The overall knee state improvement on a 7‐point Likert scale, whereby patients were classified as improved (very much better/substantially better) or not improved (a little better/no change/a little worse/substantially worse/very much worse).
− The willingness to have the surgery again (yes/no).

Surgeons completed the objective part of the Knee Society Score (KSS), ranging from 0 (worst) to 100 (best) points [18, 23]. The KSS was not available at 2 years due to the lack of routine follow‐up visits. Complications such as thromboembolic event, infection, reoperation, revision or death were recorded as adverse events. Revision was defined as reoperation to exchange part or all of the TKA.

The study was conducted following the Declaration of Helsinki and approved by the local ethics committee (reference: 2016‐01777). Written informed consent to participate was obtained from all patients.

### Statistical analysis

Descriptive statistics are presented as means and standard deviations or frequency counts and percentages. Differences between preoperative and postoperative data were tested using paired *t* tests. Differences between groups were measured by unpaired *t* tests, Mann–Whitney *U* tests or *χ* ^2^ tests as appropriate.

**Figure 1:**
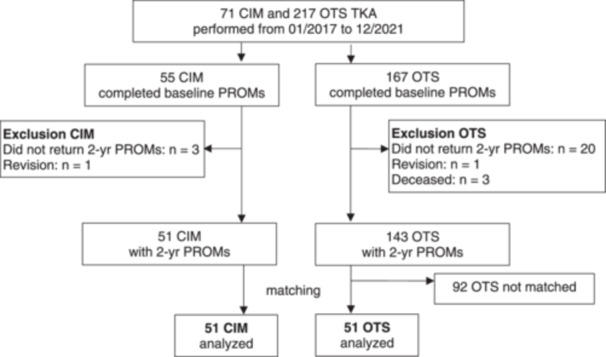
Flow chart of recruitment. CIM, customised individually made; PROM, patient‐reported outcome measure; OTS, off‐the‐shelf; TKA, total knee arthroplasty.

The a priori power calculation based on a mean effect size of 0.5 resulted in a sample size of 51 TKAs per group to assure a power of 0.8 with a one‐sided *⍺* of 0.05. To reduce the bias of a nonrandomised study and to adjust for differences in patient characteristics, we performed a propensity score matching based on the variables age, body mass index, sex, KL grade and ASA score. From 194 TKA with PROMs at 2 years available, we matched 51 CIM to 51 OTS TKA (Figure 1).

Statistical analyses were performed using IBM SPSS statistics for Windows, Version 29.0.1.0, IBM Corp and R, Version 4.1.3 [24]. Matching was performed with the MatchIt package in R, Version 4.5.3.

## RESULTS

### Recruitment and baseline measures

We analysed data from 51 CIM TKA (40 patients, 17 [43%] women) and 51 OTS TKA (48 patients, 21 [44%] women) (Figure 1). Patient characteristics are described in Table 1. Patients with CIM TKA were more likely to have supplementary insurance, which is required in Switzerland to cover the cost of CIM TKA (Table 1). PROMs were higher in CIM TKA at baseline, indicating better subjective function. The KSS was lower in CIM TKA, indicating poorer objective function (Table 1).

**Table 1:** Patient characteristics and baseline measures.

	CIM (*n* = 51)	OTS (*n* = 51)	Difference, *p* [95% CI]
*Patients characteristics*			
Age (years)	67 (±8)	67 (±10)	.896 [−3 to 4]
BMI (kg/m^2^)	28 (±6)	28 (±6)	.611 [−3 to 2]
Sex (women)	22 (43%)	22 (43%)	.999
Insurance			<.001
Basic	5 (10%)	35 (69%)	
Supplementary	46 (90%)	16 (31%)	
Side, left	22 (57%)	31 (61%)	.112
Bilateral surgery	22 (43%)	6 (12%)	<.001
KL grade			.999
3	2 (4%)	4 (8%)	
4	49 (96%)	47 (92%)	
ASA classification			.640
I/II	48 (94%)	47 (92%)	
III	3 (6%)	4 (8%)	
Length of stay (days)	6 (±1)	6 (±1)	.241 [0 to 1]
*Baseline measures*			
FJS‐12	21 (±16)	14 (±10)	.005 [2 to 13]
HAAS (not administered)			
KOOS symptoms	46 (±17)	42 (±17)	.226 [−3 to 11]
KOOS pain	48 (±16)	40 (±14)	.009 [2 to 14]
KOOS daily living	59 (±16)	47 (±17)	<.001 [6 to 18]
KOOS sports	24 (±18)	17 (±15)	.050 [0 to 13]
KOOS quality of life	29 (±16)	23 (±13)	.035 [0 to 12]
EQ‐5D‐3L	0.70 (±0.14)	0.58 (±0.19)	<.001 [0.05 to 0.18]
EQ‐VAS	72 (±16)	57 (±21)	<.001 [7 to 22]
KSS	44 (±12)	56 (±16)	<.001 [7 to 18]

*Note*: Data shown as mean (±SD)/*n* (%).

Abbreviations: ASA, American Society of Anesthesiologists; BMI, body mass index; CI, confidence interval; CIM, customised individually made; FJS‐12, Forgotten Joint Score; HAAS, High‐Activity Arthroplasty Score; KL, Kellgren and Lawrence grade of osteoarthritis; KOOS, Knee injury and Osteoarthritis Outcome Score; KSS, Knee Society Score; *n*, number of patients; OTS, off‐the‐shelf; SD, standard deviation; VAS, Visual Analogue Scale.

### Postoperative PROMs

**Figure 2:**
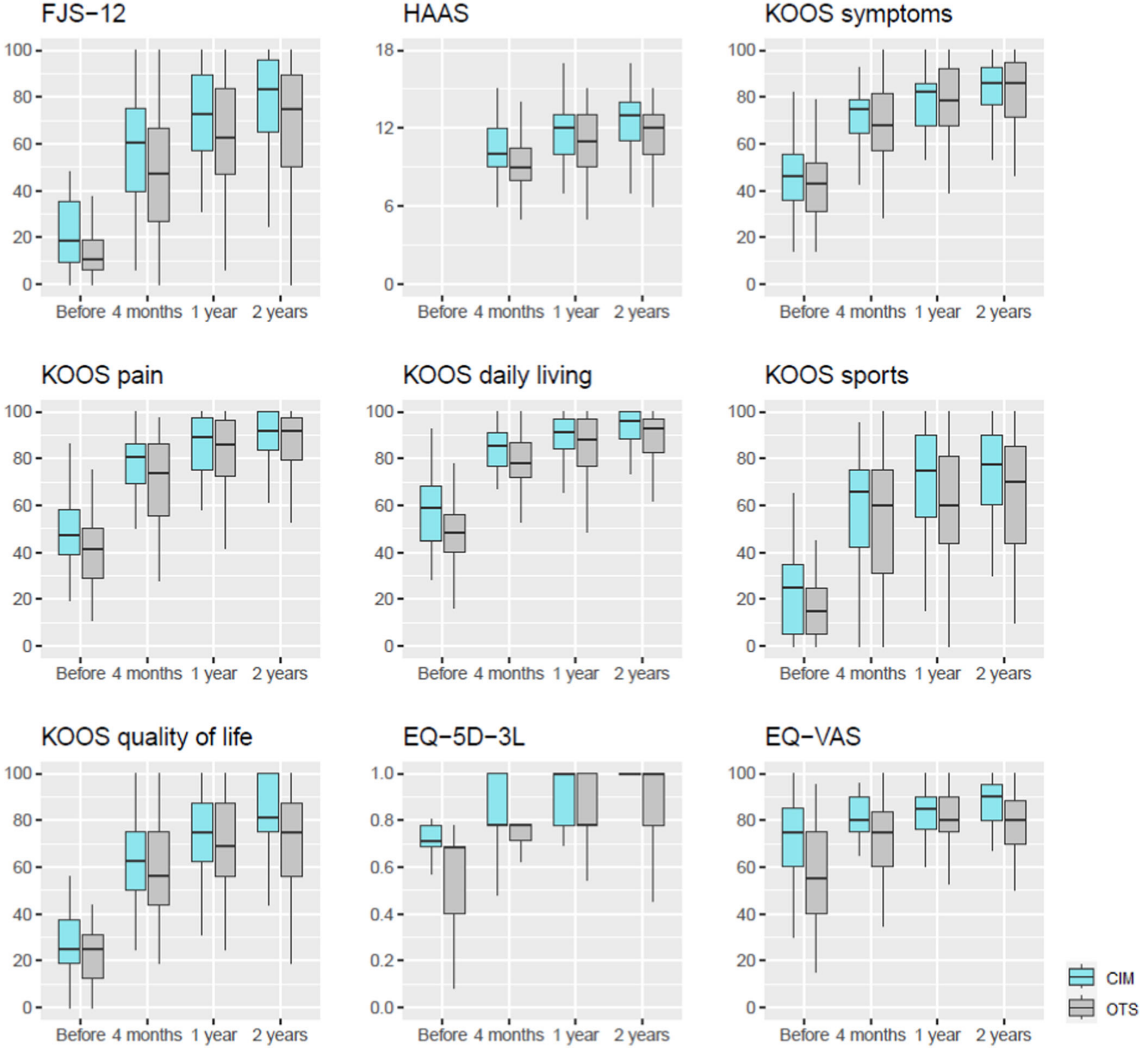
Boxplots of patient‐reported outcome measures, customised individually made (CIM) compared to off‐the‐shelf (OTS) total knee arthroplasty (TKA) patients. FJS‐12, Forgotten Joint Score; HAAS, High‐Activity Arthroplasty Score; KOOS, Knee injury and Osteoarthritis Outcome Score; VAS, Visual Analogue Scale.

**Figure 3:**
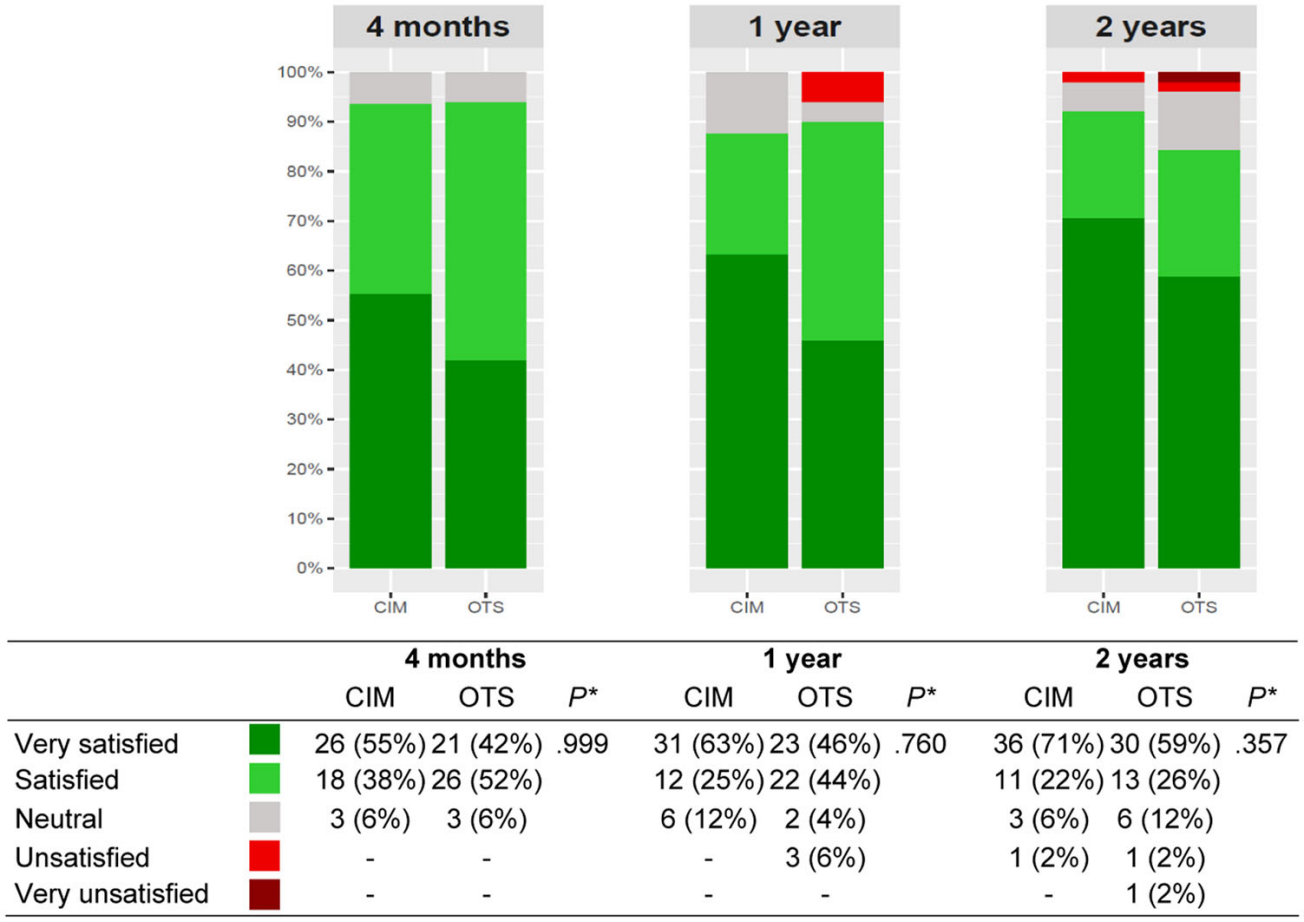
Patient satisfaction at follow‐up. CIM, customised individually made; OTS, off‐the‐shelf. Data shown as *n* (%), *Differences in satisfaction dichotomised into satisfied and not satisfied patients.

When comparing patients with CIM and OTS TKA at 2 years, the FJS‐12, HAAS, KOOS daily living, KOOS sport, KOOS quality of life and the EQ‐5D were higher for CIM TKA (*p* ≤ .058, Table 2 and Figure 2). At 2 years, 93% of CIM TKA and 85% of OTS TKA were satisfied (*p* = .357, Figure 3).

**Table 2:** Postoperative outcome measures of patients with CIM and OTS TKA.

	4 months			1 year			2 years		
	CIM (*n* = 51)	OTS (*n* = 51)	Difference, *p* [95% CI]	CIM (*n* = 51)	OTS (n = 51)	Difference, *p* [95% CI]	CIM (n = 51)	OTS (*n* = 51)	Difference, *p* [95% CI]
FJS‐12	58 (±25)	47 (±25)	.030 [1 to 21]	73 (±20)	63 (±26)	.028 [1 to 20]	77 (±23)	67 (±28)	.058 [0 to 20]
HAAS	10 (±2)	9 (±3)	.095 [0 to 2]	12 (±2)	11 (±3)	.012 [0 to 2]	13 (±2)	11 (±3)	<.001 [1 to 3]
KOOS symptoms	69 (±15)	68 (±18)	.793 [−6 to 8]	78 (±12)	78 (±16)	.941 [−5 to 6]	84 (±12)	81 (±16)	.371 [−3 to 8]
KOOS pain	78 (±13)	71 (±18)	.030 [1 to 13]	86 (±12)	82 (±17)	.197 [−2 to 10]	89 (±12)	85 (±17)	.180 [−2 to 10]
KOOS daily living	82 (±13)	77 (±15)	.050 [0 to 11]	89 (±10)	84 (±15)	.047 [0 to 11]	92 (±10)	8 6(±17)	.029 [1 to 12]
KOOS sports	56 (±27)	54 (±29)	.744 [−10 to 14]	70 (±23)	59 (±27)	.055 [0 to 21]	76 (±19)	65 (±25)	.019 [2 to 0]
KOOS quality of life	61 (±22)	59 (±23)	.621 [−7 to 11]	74 (±18)	68 (±21)	.140 [−2 to 14]	81 (±18)	71 (±24)	.028 [1 to 18]
EQ‐5D‐3L	0.83 ±0.13)	0.80(±0.12)	.163 [0.01 to .09]	0.92 (±0.11)	0.87 (±0.12)	.036 [0 to 0.10]	0.95 (±0.10)	0.90 (±0.13)	.030 [0 to .09]
EQ‐VAS	79 (±14)	71(±19)	.018 [1 to 15]	84 (±10)	78 (±15)	.030 [1 to 11]	87 (±10)	7 (±16)	<.001 [4 to 15]
Satisfaction	44 (94%)	47 (94%)	.999	43 (88%)	45 (90%)	.760	47 (92%)	43 (84%)	.357
Improvement	38 (81%)	39 (89%)	.388	45 (92%)	43 (90%)	.740	48 (94%)	45 (90%)	.487
Surgery again	44 (98%)	44 (98%)	.999	44 (96%)	45 (96%)	.999	49 (96%)	47 (98%)	.999
KSS	91 (±7)	83 (±9)	<.001 [5 to 12]	94 (±4)	87 (±8)	<.001 [4 to 10]			

*Note*: Data shown as mean (±SD)/*n* (%).

Abbreviations: CI, confidence interval; CIM, customised individually made; FJS‐12, Forgotten Joint Score; HAAS, High‐Activity Arthroplasty Score; KOOS, Knee injury and Osteoarthritis Outcome Score; KSS, Knee Society Score; *n*, number of patients; OTS, off‐the‐shelf; SD, standard deviation; VAS, Visual Analogue Scale.

For all patients, all PROMs improved from baseline to each follow‐up (data not shown, *p* < .001), from 4 months to 1 year (*p* < .001) and from 4 months to 2 years (*p* < .001). From 1 year to 2 years, all PROMs improved (*p* < .034), besides the EQ‐VAS (*p* = .906). When comparing the change scores of patients with CIM and OTS TKA, we found no group differences except for the EQ‐VAS change at 1 year, which was higher for OTS TKA (*p* = .027, additional material: Table 3).

**Table 3:** Postoperative change in outcome measures of patients with CIM and OTS TKA.

	Change baseline to 4 months			Change baseline to 1 year			Change baseline to 2 years		
	CIM (*n* = 51)	OTS (*n* = 51)	Difference, *p* [95% CI]	CIM (*n* = 51)	OTS (*n* = 51)	Difference, *p* [95% CI]	CIM (*n* = 51)	OTS (*n* = 51)	Difference, *p* [95% CI]
FJS‐12	37 (±25)	32 (±25)	.283 [−5 to 16]	51 (±23)	48 (±27)	.513 [−7 to 13]	55 (±26)	52 (±27)	.595 [−8 to 13]
KOOS symptoms	22 (±21)	25 (±24)	.517 [−12 to 6]	32 (±19)	35 (±21)	.517 [−11 to 5]	37 (±19)	39 (±20)	.694 [−9 to 6]
KOOS pain	30 (±19)	31 (±20)	.897 [−8 to 7]	37 (±19)	42 (±18)	.293 [−11 to 3]	41 (±20)	45 (±18)	.283 [−11 to 3]
KOOS daily living	24 (±20)	29 (±19)	.178 [−13 to 3]	30 (±18)	36 (±20)	.093 [−14 to 1]	34 (±18)	39 (±19)	.144 [−12 to 2]
KOOS sports	33 (±26)	37 (±31)	.466 [−16 to 8]	45 (±26)	44 (±28)	.813 [−10 to 13]	52 (±21)	50 (±28)	.671 [−8 to 12]
KOOS quality of life	33 (±26)	36 (±26)	.538 [−14 to 7]	44 (±24)	46 (±24)	.825 [−11 to 9]	52 (±24)	48 (±27)	.485 [−7 to 14]
EQ‐5D‐3L	0.14 (±0.19)	0.21 (±0.22)	.107 [−0.15 to 0.15]	0.22 (±0.18)	0.28 (±0.23)	.183 [−0.14 to 0.03]	0.25 (±0.18)	0.32 (±0.23)	.121 [−0.14 to .02]
EQ‐VAS	8 (±20)	13 (±26)	.291 [−14 to 4]	11 (±18)	21 (±23)	.027 [−17 to −1]	15 (±20)	19 (±24)	.336 [−13 to 4]
KSS	48 (±13)	25 (±15)	<.001 [17–29]	50 (±13)	30 (±14)	<.001 [14 to 26]			

*Note*: Data shown as mean (±SD)/*n* (%).

Abbreviations: CI, confidence interval; CIM, customised individually made; FJS‐12, Forgotten Joint Score; KOOS, Knee injury and Osteoarthritis Outcome Score; KSS, Knee Society Score; OTS, off‐the‐shelf; *n*, number of patients; SD, standard deviation; VAS, Visual Analogue Scale.

### Postoperative KSS

When comparing patients with CIM and OTS TKA, the KSS was higher for CIM TKA at 4 months and 1 year (Table 2). The KSS improved for all patients from baseline to 4 months (data not shown, *p* < .001), from baseline to 1 year (*p* < .001) and from 4 months to 1 year (*p* < .001). When comparing the change scores of patients with CIM and OTS TKA, we found a higher change for CIM TKA from baseline to 4 months (*p* < .001) and from baseline to 1 year (*p *< .001, additional material: Table 3).

### Adverse events

During follow‐up, three patients with OTS TKA died unrelated to TKA. One patient with CIM TKA (at 13 months) and one patient with OTS TKA (at 9 months) required complete revision. The revision rate was 1.4% (1 of 71) for CIM and 0.5% (1 of 217) for OTS TKA (*p* = .433). Three patients with CIM and 20 patients with OTS TKA did not complete the PROMs at 2 years. These patients were excluded from the analysis (Figure 1). No further adverse events occurred in the patients included in the final analyses.

## DISCUSSION

The most important finding was that PROMs were better for patients with CIM TKA at 2 years, namely the FJS‐12, HAAS, KOOS daily living, KOOS sport, KOOS quality of life and EQ‐5D. Patient satisfaction was higher for CIM TKA, but there was no significant difference in satisfaction between CIM and OTS TKA. Thus, our hypothesis was partially confirmed. Patients with CIM TKA showed better functional and health‐related quality of life outcomes but were not superior regarding satisfaction at 2 years.

Our results in CIM TKA patients are comparable to those of other studies that have reported results after CIM TKA with the ORIGIN® prosthesis [7, 10, 22, 25]. However, no other comparative study results are currently available. A very recent study using the same implant and alignment strategy reported the FJS‐12 and KOOS of 143 CIM TKA. At a mean follow‐up of 2.8 years, the FJS‐12 was 69, KOOS symptoms 82, pain 85, daily living 83, sports 52 and quality of life 75 [10]. The mean FJS‐12 was thus eight points lower than in our study. The satisfaction rate of 94% was slightly higher than the 92% we found at 2 years. Another recent study reported a large improvement in KSS at 1‐year follow‐up in a series of 266 CIM TKA. The final KSS of 94 was similar to our result, unfortunately PROMs were not reported [25]. The same research group also found very good results in 37 CIM TKA with previous osteotomies and/or extra‐articular fracture sequelae [7]. At a mean follow‐up of 1.3 years, the FJS‐12 was 66, KOOS symptoms 75, pain 87, daily living 87, sports 59 and quality of life 72 [7]. Our own research group found promising initial results in the first series of 25 CIM TKA [22]. At 1 year, the FJS‐12 was 73, KOOS symptoms 80, pain 86, daily living 87, sports 64 and quality of life 73. The KSS was also reported at 94 points [22]. We were able to confirm our findings, as the results in this study were very similar at 1 year.

Focusing on the FJS‐12, the CIM TKA results were very good and exceeded the results of studies using the same CIM TKA implant [7, 10] or studies focusing on kinematic alignment [28]. Regarding OTS TKA, we found one study that measured the FJS‐12 in patients with a computer‐navigated Attune CR at 1 year [4]. With a mean FJS‐12 of 68, the result was slightly better than our mean FJS‐12 of 63.

Regarding patient satisfaction, we found only 2% unsatisfied patients in the CIM TKA group and 4% in the OTS TKA group (combined: 3%). Although we found no statistical differences between the two groups, this is significantly lower than what was reported in a previous systematic review, which found 10% unsatisfied patients [8]. Of course, our relatively small number of patients must be taken into account. However, our results are consistent with a larger CIM TKA study that also found only 3% unsatisfied patients [10]. When discussing patient satisfaction, it is important to note that there is no consensus on the best way to measure it. The concept of patient satisfaction is subjective and heterogeneous, making it difficult to assess [20]. There is no gold standard for measuring patient satisfaction, and quantifying it in a valid way is challenging [21]. In this study, we used a 5‐point Likert scale, along with other validated PROMs, but found no statistical difference between CIM and OTS TKA.

In a previous comparison of results between OTS TKA (Attune CR) and CIM TKA (Conformis iTotal® CR G2, Conformis Inc.), only minor clinically relevant advantages for CIM TKA were found at 2 years, such as a higher HAAS for the CIM TKA [35]. In this recent study, however, in which not only the individual shape of each patient's knee was personalised but also a personalised alignment strategy was applied, clinically relevant differences in several PROMs were found in favour of the CIM TKA. This is even more remarkable given that the baseline scores for CIM TKA were higher than those for OTS TKA. One might have assumed that improvement would be difficult to achieve because patients subjectively felt less restricted. However, we did not find this and instead found a significant improvement in CIM TKA compared to OTS TKA. Furthermore, no ceiling effects were found in any of the PROMs (Figure 2), with the exception of the EQ‐5D, and this problem is known to occur in patients with TKA [37].

This is the first study to compare the PROMs of patients with CIM TKA (using the ORIGIN® prosthesis) versus OTS TKA. The strength of our study is the prospective matched‐pair design using a comprehensive set of PROMs over multiple follow‐ups. Nevertheless, our study has several limitations. We cannot exclude the possibility of selection bias due to the lack of randomisation, which was not possible in our setting. To limit selection bias, we performed propensity score matching. Although we consecutively asked all our TKA patients to complete PROMs preoperatively, only 77% did so. In addition, the loss to follow‐up of patients who did not return their 2‐year PROMs questionnaire was 10%. Despite great efforts to achieve a high response rate, attrition bias cannot be excluded. The patients were recruited from a private hospital in Switzerland, and, therefore, the study results may not be fully generalisable. At the time of the study, the PS model of the ORIGIN® prosthesis was the only one available, as the ORIGIN® CR was introduced in 2022. Therefore, it was technically unavoidable to compare the ORIGIN® PS with the Attune CR. In both centres, this was the main implant used at the time. Further comparative studies, especially randomised controlled trials, are needed to confirm and generalise our findings.

## CONCLUSIONS

The current study found that CIM TKA patients had better functional outcomes at 2 years as measured by the FJS‐12, HAAS, KOOS daily living, KOOS sport, KOOS quality of life and EQ‐5D. Patient satisfaction was high and did not differ from OTS TKA patients. Knee surgeons are used to about one in five patients being not satisfied with the outcome of TKA at 1 or 2 years. It is clinically relevant that for CIM TKA, this rate is shown to be closer to one in 15 patients.

## AUTHOR CONTRIBUTIONS

Nicole Vogel, Raphael Kaelin and Markus P. Arnold designed the study. Nicole Vogel, Raphael Kaelin and Markus P. Arnold contributed to data collection. Nicole Vogel coordinated the study, analysed the data and drafted the manuscript. Nicole Vogel, Raphael Kaelin and Markus P. Arnold revised the final manuscript.

## CONFLICT OF INTEREST STATEMENT

Markus P. Arnold is a consultant for Symbios. The remaining authors declare no conflict of interest.

## ETHICS STATEMENT

The study was approved by the local ethics committee (ID: 2016‐01777) and written informed consent was obtained from all patients in the study.
